# Sweet reward increases implicit discrimination of similar odors

**DOI:** 10.3389/fnbeh.2014.00158

**Published:** 2014-05-06

**Authors:** Eva Pool, Sylvain Delplanque, Christelle Porcherot, Tatiana Jenkins, Isabelle Cayeux, David Sander

**Affiliations:** ^1^Swiss Center for Affective Sciences, University of Geneva-CISAGeneva, Switzerland; ^2^Laboratory for the Study of Emotion Elicitation and Expression, Department of Psychology, FPSE, University of GenevaGeneva, Switzerland; ^3^Firmenich, SAGeneva, Switzerland

**Keywords:** reward, odor discrimination, emotional learning, perceptual representation, incentive salience, taste-odor conditioning

## Abstract

Stimuli associated with emotional events signal the presence of potentially relevant situations, thus learning to rapidly identify this kind of stimuli can be highly beneficial. It has been demonstrated that individuals acquire a better perceptual representation of stimuli associated with negative and threatening emotional events. Here we investigated whether the same process occurs for stimuli associated with positive and rewarding emotional events. We used an appetitive Pavlovian conditioning paradigm during which one of two perceptually non-distinguishable odors was associated with a rewarding taste (i.e., chocolate). We investigated whether appetitive conditioning could improve the recognition of the odor associated with the reward, rendering it discriminable from its similar version that was never associated with the reward. Results revealed a dissociation between explicit perception and physiological reactions. Although participants were not able to explicitly perceive a difference, they reacted faster, inhaled more and had higher skin conductance responses when confronted with the reward-associated odor compared to its similar version that was never associated with the reward. Our findings have demonstrated that positive emotional associations can improve the implicit perceptual representation of odors, by triggering different physiological responses to odors that do not seem to be otherwise distinguishable.

## Introduction

Understanding how organisms deal with their limited attentional resources in an environment composed of a virtually infinite number of stimuli has always been of main interest for cognitive sciences (Posner, 1980). For instance, if two persons are chatting and watching their children on a playground, they will not be able to represent every action their children make, because a part of their resources is invested in the conversation. Nonetheless, some particular actions such as the children crying or calling for them will be represented in spite of the conversation. Why is the processing of those particular stimuli privileged? It has been proposed that emotional stimuli may have a facilitated access to an organism’s attentional resources (Vuilleumier, 2005).

A large amount of evidence has shown that emotional stimuli are difficult to ignore (e.g., Segerstrom, 2001; Compton et al., 2003); that our gaze is rapidly oriented toward them (e.g., Nummenmaa et al., 2009; Theeuwes and Belopolsky, 2012); that disengaging attention from them is hard (e.g., Fox et al., 2001; Yiend and Mathews, 2001; di Pellegrino et al., 2011); and that in a complex environment with competing stimuli, they have a prioritized access to attentional resources (e.g., Ohman et al., 2001; Anderson, 2005; Hodsoll et al., 2011). These effects of emotional stimuli on cognitive processing have been shown to be at least mediated by an enhancement of the neuronal activity linked to sensorial processing. Indeed, neuroscientific evidence strongly suggests that sensorial information is processed more efficiently if it has emotional content (see Phelps and Ledoux, 2005; Vuilleumier, 2005, for a review). More particularly, the activity linked to sensory processing at very early stages is amplified during the processing of emotional stimuli, as demonstrated with electroencephalography, (e.g., Pourtois et al., 2004; Brosch et al., 2008; Hickey et al., 2010) and functional imagery (e.g., Vuilleumier et al., 2001; Grandjean et al., 2005; Mohanty et al., 2008).

The degree of specificity of this emotional facilitation remains to be fully understood. Is the perceptual enhancement selective for a particular emotional stimulus or does it generalize to neutral stimuli with similar perceptual characteristics? If it is specific, the discrimination between an emotional stimulus and a perceptually similar but neutral stimulus should be easier compared to the discrimination between two neutral stimuli. Li et al. (2008) investigated these questions for the processing of negative threatening stimuli by using a triangular test to measure the discriminability of similar odors (i.e., enantiomers) combined with an aversive emotional learning paradigm. The triangular test is a forced choice procedure used to investigate perceptual discriminability, in which a stimulus is presented twice with a third different but similar stimulus that has to be identified (Laska and Teubner, 1999). Through this procedure Li et al. (2008) demonstrated that after aversive emotional learning, during which one of the two odors was associated with an unpleasant electric shock, two odors that were initially indistinguishable became discriminable. The enhancement of the sensorial abilities through aversive emotional learning seems to thus be highly selective, when the learning context requires high discrimination abilities. This mechanism could be highly adaptive, because it allows the organism to discriminate stimuli with an emotional meaning from other less meaningful but similar stimuli and thereby preventing the organism to over-react when it is not necessary. To the best of our knowledge, this mechanism has never been tested for positive emotional stimuli. Positive and negative emotional stimuli should have the same influence on the sensorial processing, because the privileged status of the emotional stimuli would depend on the affective relevance of the stimulus for the concerns of the individual perceiving it, rather than on a distinct valence specific mechanism (Frijda, 1988; Sander et al., 2005). If an individual learns to associate an intrinsically neutral stimulus to an emotional event, either a threat or a reward, the stimulus that was initially neutral but acquired affective relevance through the learning experience also gains a privileged access to the individual resources (for aversive learning see Alpers et al., 2005; Koster et al., 2005; for appetitive learning see Seitz et al., 2009; Austin and Duka, 2010; Hickey et al., 2010; Anderson et al., 2011; Notebaert et al., 2011; Pool et al., 2014).

Based on this assumption, we investigated whether (i) discrimination capabilities could be enhanced through an appetitive emotional learning using positive rewarding stimuli and, if any, (ii) the degree of the selectivity of this enhancement at an implicit and an explicit level. To do so, we adapted and modified the experimental procedure used by Li et al. (2008), by combining a triangular test in which participants were asked to distinguish between two similar odors with an appetitive emotional learning paradigm. We selected two pairs of indistinguishable odors as the conditioned stimuli in a Pavlovian conditioning task. For the first pair of odors, one of them was associated with a rewarding piece of chocolate (Positive Conditioned Stimulus; CS+) whereas its similar version was not (CS+ similar). For the second pair, neither odor was associated with the pleasant reward (Negative Conditioned Stimulus; CS- and CS-similar). If positive and negative stimuli possess similar reinforcing capabilities then the odor that acquired affective relevance by being associated with a reward should be better discriminated from the similar odor compared to the odors that have never been associated with the reward. During the triangular test, two different measures of odor discrimination were used. The first was an explicit and classical measure consisting in the accuracy of the behavioral choices (Laska and Teubner, 1999; Li et al., 2008), the second was an implicit measure consisting in the inspiration volume (Bensafi et al., 2003, 2007; Frank et al., 2003; Mainland and Sobel, 2006). The inspiration volume was used as an implicit measure for two reasons. First, it is modulated by the pleasantness of the odor (Bensafi et al., 2003; Frank et al., 2003); since in appetitive conditioning the value of the reward is transferred to the CS, the odor associated with the reward should become more pleasant (De Houwer et al., 2001) and consequently, the inspiration volume could be modulated. Second, the inspiration volume is not influenced by any conscious strategy (Bensafi et al., 2007). We predicted that the odor that had acquired affective relevance by being associated with the reward would be discriminated from its pair: (a) explicitly as reflected by a higher accuracy in the behavioral choices during the triangular test after conditioning and (b) implicitly as reflected by a bigger inspiration volume for the reward-associated odor than for its similar version.

## Materials and methods

### Participants

Eighteen participants who liked chocolate and were not dieting were recruited on the premises of the University of Geneva. They received 20 Swiss francs for their participation. Two participants were later excluded due to a technical problem during the physiological recordings. The 16 participants included in the analysis (5 males; 26 ± 3.22 years old) had no problems with odor perception and were not wearing any fragrance. Participants were asked to refrain from eating 4 h before the experimental session to increase their motivation to eat chocolate.

### Materials

#### Stimuli

Two different pairs of odors were used (Firmenich, SA, Geneva, Switzerland). Each pair was composed by an odor that was qualitatively coherent in a feeding context and its similar version. This latter was created by mixing the original odor with a small percentage of *Hedion*® (methyl dihydrojasmonate), a fresh jasmin like odor. More specifically, first pair was composed of a *lemon* odor (100%) and a *lemon-Hedion* blend odor (98% and 2% respectively), the second of a *strawberry* odor (100%) and a *strawberry-Hedion* blend odor (97% and 3% respectively). These odors were selected based on a previous pilot study (*N* = 20) that revealed that when participants were asked to distinguish an odor mixed with *Hedion* from its pure version, their performances were not significantly better than chance, *t*_(19)_ = −0.85, *p =* 0.430. The pure *Hedion* was also used to control for its perceptibility. Odors were administered through a computer-controlled olfactometer with an air flow fixed at 1.5 L/min that delivered the olfactory stimulation rapidly, without thermal and tactile confounds (Pool et al., 2014) via a nasal cannula.

The rewarding chocolate consisted of a small 0.5 g piece of chocolate (dark or milk, according to the participants’ preferences) to prevent that satiation processes modified the chocolate’s rewarding value during the conditioning procedure (Small et al., 2001).

#### Physiological recordings

Respiration and electrodermal activities were recorded using the MP150 Biopac Systems (Santa Barbara, CA) with a 1000 Hz sampling rate. The respiratory activity was recorded through a 2.5 mm tube (interior diameter) positioned at the entrance of the participants’ right nostril, on the nasal cannula used to deliver the odorants, and connected to a differential pressure transducer (TSD160A; ± 2.5 cm H_2_O sensitivity range) to continuously recorded variations in the nostril airflow. The signal was first low-pass filtered at 1 Hz and the duration of the inspiration was calculated as the length of the depression (in ms) between two consecutive crossing of the zero values after the stimulus onset. The integral of flow variations and its maximal value within this duration were also calculated for each trial.

Electrodermal activity was measured with Beckman Ag–AgCl electrodes (8-mm diameter active area) filled with a skin conductance paste (Biopac) attached to the palmar side of the middle phalanges of the second and third fingers of the participants’ nondominant hand. Specific skin conductance responses (SCRs) were measured in microSiemens and analyzed offline (Bandpass filter: 0.05–5 Hz). They were scored as changes in conductance starting in the 1- to 5-s interval after the beginning of the stimulus. SCRs were square root transformed to normalize the data (Dawson et al., 2000).

### Procedure

Before the experiment started, physiological sensors were positioned on the participants. They also indicated whether they preferred dark (*N* = 9) or milk chocolate (*N* = 7) so that the reward used in the Pavlovian conditioning could be consequently adapted. Subsequently, they completed a control triangular test to measure their *Hedion* perception. They then completed the experimental procedure consisting in four triangular tests, two before and two after the Pavlovian conditioning. At the end of the experiment, they answered questions concerning the manipulation check. The entire experimental session took around 75 min.

#### Pavlovian conditioning

Each pair of odors was assigned to the Pavlovian role of the “CS+” or “CS−”. For the CS+ pair, one of the two odors was associated with a rewarding taste of chocolate (CS+) whereas the other one was not (similar CS+). For the “CS−”, neither of the two odors was associated with a reward (CS−; similar CS−). The assignment of the odors to the Pavlovian roles was counterbalanced across participants. For the sample included in the analysis, all odors were used as CS+ for at least 3 participants: the lemon (*N* = 5), the lemon-*Hedion* (*N* = 5), the strawberry (*N* = 3) and the strawberry-*Hedion* (*N* = 3).

**Figure 1:**
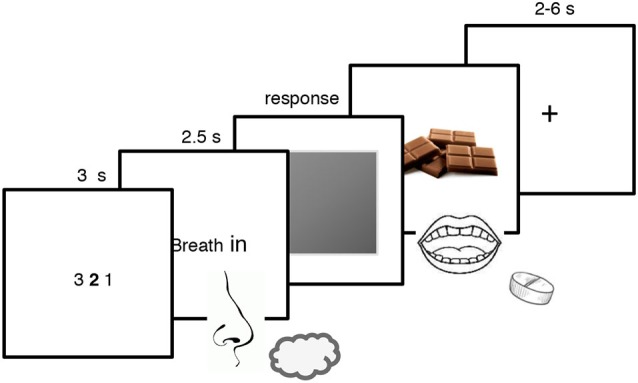
**Pavlovian conditioning paradigm used in the present experiment.** Participants were exposed to two pairs of odors, each composed of one odor and its similar version which was created by blending it with *Hedion* (CS+; similar CS+ and CS−; similar CS−). They smelled the odor and pressed on a keyboard to remove the gray patch and discovered whether the odor was associated with the rewarding chocolate or not. The CS+ was associated with the reward whereas the similar CS+, the CS− and the similar CS− were not.

There were 18 trials for each odor for a total of 72 trials. Every trial began with a 3 s countdown followed by an inspiration cue that request the participant to breath in evenly and trigger the release of the odor for 2.5 s. A gray patch then appeared and participants were asked to press as quickly as possible the “x” key to remove the patch and reveal a picture of chocolate for the CS+ and a red cross for all the other stimuli (i.e., the similar CS+, the CS− and the similar CS−). They were also explicitly asked to guess whether a particular odor could predict the rewarding chocolate delivery. When the chocolate picture was displayed on the screen, participants ate a small piece of chocolate and drank a sip of water (Prévost et al., 2012). To underline that the reward delivery depended on the odor and not on their action, participants were told that the key-pressing task was a measure of their sustained attention, independent of the odor-reward contingencies (Talmi et al., 2008). They were also informed that not responding during the 1-s interval after the gray patch onset would trigger the next screen anyway (Talmi et al., 2008). Every trial ended with an inter-trial interval of 4–6 s for the non-rewarded trials and of 2–4 s for the rewarded trials (see Figure 1). The Pavlovian conditioning procedure lasted around 30 min.

After the conditioning task, participants took a small break (1–2 min) and then they evaluated on a visual analog scale presented on the screen, the subjective pleasantness (from “extremely unpleasant” to “extremely pleasant”), intensity (from “not perceived” to “extremely strong”) and familiarity (from “not familiar at all” to “extremely familiar”) of each odor and of the *Hedion* odor alone (e.g., Delplanque et al., 2008). The odor evaluation procedure lasted about 5 min.

#### Triangular test

We created a computerized version of the triangular discrimination test designed by Laska and Teubner (1999). Three pictures of bottles associated with three different odors were presented on the screen. Participants were told that two of the odors were identical and one they had to identify was different.

Each trial began with a screen displaying the first bottle pointed by an arrow for 2–5 s, then a 3 s countdown began followed by an inspiration cue and the odor was delivered for 1 s. Afterwards, the bottle remained on the screen for 8–5 s (so that the inter-stimulus interval was 10 s in total). Then, the second and the third bottle appeared and the same procedure was repeated. Once the three odors had been smelled, participants were instructed to use the mouse to determine the bottle containing the odd odor by clicking on the corresponding picture of bottle. During the inter-trial interval participants were asked to wait for 15 s. All possible combinations of apparition of the three bottles (and odors; i.e., 6 in total) were presented to each and randomly across participants. Separate triangular tests were administered for each pair of odors (lemon and lemon-*Hedion* blend; strawberry and strawberry-*Hedion* blend) used in the conditioning. To accomplish a triangular test, participants took around 6 min.

At the beginning of the experiment, participants accomplished a control triangular test in which they were asked to discriminate *Hedion* from odorless air.

#### Manipulation check

Participants were asked to eat a piece of the chocolate that was used during the conditioning and to evaluate, on a visual analog scale presented on the screen, its subjective pleasantness (from “extremely unpleasant” to “extremely pleasant”), intensity (from “extremely weak” to “extremely strong”) and familiarity (from “never tasted before this experiment” to “extremely familiar”). Subsequently, they answered two questions about chocolate: one about motivation (i.e., “On a scale from 1 to 10, how much would you say that you sometimes crave chocolate?”) and the other about hedonic pleasure (i.e., “On a scale from 1 to 10, how much would you say that you like chocolate?”; see Pool et al., 2014). This last part of the experiment took around 5 min.

## Results

### Data analysis

For the analysis of the Pavlovian conditioning and the triangular tests we used a repeated measures analysis of variance (ANOVA) and planned contrasts according to the hypothesis that was tested. Effect sizes are measured as eta squared (*η*^2^) for the repeated measure ANOVA and as Cohen’s d (*d*) for the planned contrasts.

### Manipulation Check

Participants evaluated the piece of chocolate’s taste used as reward as being pleasant (*M* = 93.16, *SD =* 8.77 out of 100); intense (*M* = 76.01; *SD =* 19.11 out of 100); and familiar (*M* = 94.53, *SD =* 6.41 out of 100), showing that the chocolate used as reward was indeed perceived as a pleasurable experience. Moreover, they reported a mean of 9.41 (*SD* = 1.01) out of 10 for the likeability item and a mean of 8.54 (*SD* = 1.29) out of 10 for the craving item on the questions about chocolate, showing that they associated chocolate with hedonic pleasure and motivation (Berridge and Robinson, 2003).

### Hedion Perceptibility

In the control triangular test, participants discriminated the *Hedion* odor from the odorless air with an accuracy of 71.87% (±23.36%) which was significantly better than the chance level of 33%, *t*_(15)_ = 7.59, *p* < 0.001, *d* = 2.6. For the odor evaluation, participants reported a mean of 44.59 (±24.46; out of 100) for the intensity scale, a mean of 59.25 (±16.81; out of 100) for the pleasantness scale and a mean of 53.87 (±19.72; out of 100) for the familiarity scale.

### Pavlovian Conditioning

To assess the success of the Pavlovian conditioning, we used the reaction time of the key-pressing task and the SCRs as implicit indexes and the likeability rating of the odors used as CSs after conditioning as an explicit index.

The reaction times of three participants could not be recorded due to technical problems. All responses that were more than three standard deviations from the mean (<1% of the trials), or absent (<3% of the trials) were removed. A planned contrast revealed a specific effect of conditioning: participants were faster when the CS+ odor (*M* = 384.08, *SD* = 25.77) was delivered than when the similar CS+, the CS− and the similar CS− odors were delivered (*M* = 405.66, *SD* = 21.53), *t*_(12)_ = 2.44,* p* = 0.031, *d* = 0.30. To test whether this difference could be accounted for in terms of reward learning, we conducted two other planned contrasts that revealed that this difference was significant in the second part of the conditioning, *t*_(12)_ = 2.62, *p* = 0.022,* d* = 0.32, but not in the first, *t*_(12)_ = 0.16, *p* = 0.860.

**Figure 2:**
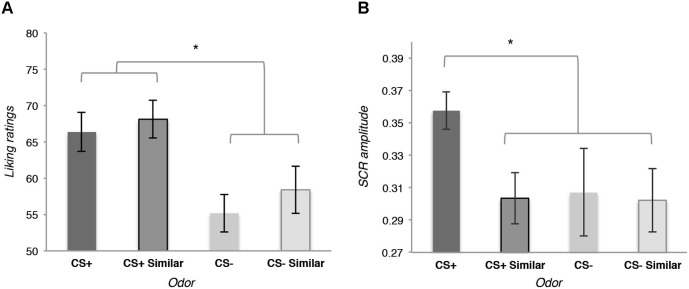
**(A)** Means of the likeability ratings for the four odors used as conditioned stimuli (CS) at the end of the Pavlovian conditioning. **(B)** Means of the amplitude of the skin conductance responses (SCR) during the perception of the four odors used as CS at the end of the Pavlovian conditioning during the rating phase. Error bars (± 1 *SEM*) are adapted for within design (Cousineau, 2005).

The likeability analysis revealed a general effect of conditioning: participants did not evaluate the CS+ odor as significantly more likeable than the similar CS+, the CS− and the similar CS−, *t*_(15)_ = 1.19, *p* = 0.252; but they globally evaluated the CS+ and the similar CS+ as being more likeable than the CS− and the similar CS−, *t*_(15)_ = 2.45, *p* = 0.026, *d* = 0.47 (see Figure 2A).

Autonomic responses recorded during the same phase were specifically affected by the conditioning: a planned contrast revealed that the amplitude of the SCR was bigger for the CS+ than for the similar CS+, the CS− and the similar CS−, *t*_(12)_ = 2.41, *p* = 0.032, *d* = 0.21^1^ (see Figure 2B).

### Triangular test

Similarly to the conditioning, results on the triangular test showed specific effects only for the implicit measure, but not for the explicit one.

**Figure 3:**
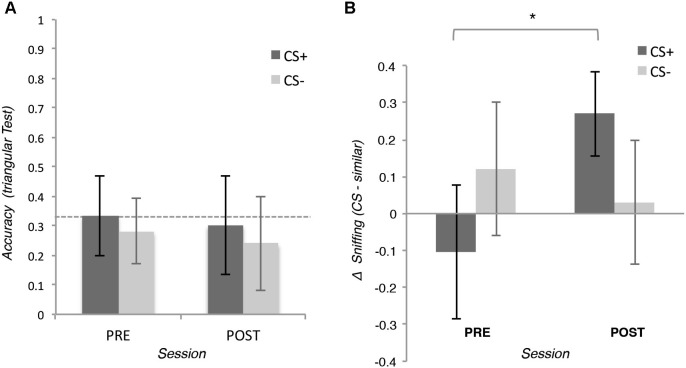
**(A)** Means of the accuracy of the behavioral choice during the triangular test in which participants were asked to discriminate two similar odors. In the positive conditioned pair (CS+), one odor was associated with the reward (CS+) whereas the other was not (similar CS+). In the negative conditioned pair, neither of the two odors (CS− and CS− similar) was associated with the reward. **(B)** Means of the integral of the volume of the inspiration during the triangular tests. Error bars (±1 *SEM*) are adapted for within design (Cousineau, 2005).

To test our hypothesis for the explicit measure, we applied a 2 (Pair: CS+ or CS− pair) × 2 (Session: pre- or post-conditioning) repeated measures ANOVA on the accuracy of behavioral choices of the triangular tests. The analysis did not reveal any significant effect (all *p_s_* > 0.05), *t*-test showed that the performance of the participants were not significantly different from the chance level of 33%, neither before nor after conditioning (all *p_s_* > 0.05; see Figure 3A).

To test our hypothesis for the implicit measures, we applied a 2 (Odor: CS+ or similar CS+) × 2 (Session: pre- or post-conditioning) repeated measures ANOVA on the integral of inspiration flow during the triangular tests. The analysis revealed a significant interaction, *F*_(15,1)_ = 5.41, *p* = 0.034, *η*^2^ = 0.26 showing that after conditioning participants inhaled more for the CS+ than for the similar CS+, *t*_(15)_ = 2.49, *p* = 0.027, *d* = 0.28, this difference was not present before conditioning, *t*_(15)_ = 0.69, *p* = 0.490. The same analysis on the CS− and the similar CS− did not reveal any significant effect (all *p_s_* > 0.05; See Figure 3B).

Since the ANOVAs revealed that conditioning increased the inspiration flow for the CS+ compared with similar CS+ but not for CS− and its similar version, we used planned comparisons to compare the inspiration across the fours odors after conditioning. Participants inhaled more when the CS+ was perceived than when the similar CS+, the CS− and the similar CS− were perceived *t*_(15)_ = 2.23, *p* = 0.041, *d* = 0.25;^2^ This difference was not present before conditioning (*p* = 0.511).

The same analysis conducted on the SCR amplitude did not reveal any significant effect (all *p* > 0.05).

## Discussion

The objective of this experiment was twofold: first to investigate whether perceptual discrimination of odors could be enhanced through appetitive emotional learning, and second, the degree of selectivity of this enhancement at an implicit and explicit level. To do so, an olfactory stimulus was associated with a sweet reward (i.e., chocolate) and we tested the capacity of the participants to discriminate it from another perceptually similar but non-conditioned stimulus. Before conditioning, the conditioned odor and its similar version were not discriminated in the triangular test, neither on the basis of accuracy of choices, nor for strength of the inspiration. After conditioning, we did not find any statistical evidence showing that participants were able to discriminate the odors in the triangular test, nonetheless their inspirations were more intense for the reward-associated odor compared to its similar non-conditioned version. A similar pattern was shown for the conditioning indexes: participants globally evaluated the reward-associated odor and its similar version more pleasurable compared with the evidently perceptually different non-conditioned odors, without differentiating the reward-associated odor from its similar non-conditioned version. However, participants’ responses to the reward-associated odor were differentiated from its similar non-conditioned version and the evidently perceptually different non-conditioned odors based on the implicit indexes of conditioning (i.e., the SCR’s amplitude after conditioning and the reaction times during conditioning). In sum, the explicit behavioral measure demonstrated that the conditioning effect of the CS+ is generalized to its similar version, whereas the physiological and implicit behavioral measure showed a specific effect of conditioning which influences selectively the CS+ but not its similar version.

Consistent with Li et al. (2008), our findings showed that emotional learning can enhance the discrimination between similar stimuli at both behavioral (reaction time) and physiological levels (inspiration and skin conductance); however by contrast Li et al. (2008) found an increase of the accuracy of the behavioral choices in the triangular test induced by aversive conditioning, while participants’ explicit discrimination was not increased in our study after the appetitive conditioning. Many factors could explain this absence of effect (e.g., number of trials, number of participants, odor choice). Nonetheless, such a dissociation between the explicit and the implicit measures is congruent with several findings in the literature.

First, the dissociation between the auto-reported evaluation of the odors’ pleasantness and the volume of the inspiration during the odor perception supports the idea that the inspiration volume is modulated by the affective properties of the odors (Bensafi et al., 2003; Frank et al., 2003; Mainland and Sobel, 2006), independently of the conscious processing (Bensafi et al., 2007). The way in which the odor is inspired can in turn influence different aspect of the odor perception such as its intensity or its identity (Mainland and Sobel, 2006). We did not find evidence showing that the increased inspiration volume improved the odor identity discrimination. However, this is congruent with the literature showing that the improvement of the odor identity is not proportional to the inspiration volume, but rather depends on the interaction between the intrinsic properties of the odor (e.g., sorption rate) and other parameters of the inspiration (e.g., velocity, Sobel et al., 1999; Mainland and Sobel, 2006).

Second, the fact that even though participants did not seem to be able to explicitly discriminate the reward-associated odor, they responded with more motivated reactions when they perceived is consistent with studies showing that it is possible to elicit affective reactions even when participants are not able to consciously report them (Winkielman and Berridge, 2004; Winkielman et al., 2005). Several theories (Murphy and Zajonc, 1993; Winkielman and Berridge, 2004; Winkielman and Schooler, 2011) postulate that the processing of affective stimuli can involve differential physiological responses and subjective feelings accessible through verbal reports. Affective processing may occur rapidly and automatically without accessing conscious processing, thereby triggering physiological responses without a parallel conscious and verbally reportable experience (Murphy and Zajonc, 1993; Silvestrini and Gendolla, 2011; Bornemann et al., 2012).

There might by an adaptive value in these differential effects of conditioning on the implicit and explicit levels. The generalization of the conditioning effects to similar stimuli at an explicit level, might allow the organism to flexibly stay vigilant to all potential indicators of reward, whereas the specificity of the conditioning effects at an implicit level might allow the organism to economize resources by reacting with expensive emotional reactions, even when positive, only when it is strictly necessary.

Finally, the increase of physiological discrimination of similar odors by appetitive emotional learning using positive rewarding stimuli is in line with studies showing an increased efficiency of sensorial processing for positive emotional stimuli (e.g., Brosch et al., 2008; Hickey et al., 2010; Hietanen and Nummenmaa, 2011) and with the prediction of appraisal theories (Frijda, 1988; Sander et al., 2005). Indeed, appraisal theories propose that both negative and positive events influence the perceptual system because they are underlain by an affective relevance mechanism rather than distinct valence-specific mechanisms. Whereas neutral stimuli associated with a threat acquired affective relevance because they increase the chances of avoiding a danger, neutral stimuli associated with a reward acquired affective relevance because they increase the chances of satisfying a goal.

## Conclusions

The findings of this experiment suggest that emotional learning using positive rewarding stimuli can increase implicit discrimination of perceptually similar odors. The ability to learn to precisely identify stimuli that are affectively relevant not only increases the chances to indeed react to emotional events, but it also reduces the chances of reacting with expensive emotional reactions when it is not necessary. For instance, if two persons are watching their children and chatting, they will intensively react specifically to the voice of their children calling them, and not to all the voices of other children calling their parents. Furthermore, our findings suggest reward-associated odors can trigger affective reactions, even when it seems that they cannot be consciously discriminated. This deserves to be further investigated since it opens an interesting window to the influence that an odor can have on our experience.

## Conflict of interest statement

The authors declare that the research was conducted in the absence of any commercial or financial relationships that could be construed as a potential conflict of interest.

## Footnotes

^1^Three participants were excluded from the analysis for missing data (listwise analysis). The maximal peak of their skin conductance appeared before the 1 s delay after the stimulus on set on at least one of the four conditions (e.g., CS+, CS+ similar, CS−, CS− similar). Therefore we could not consider these responses as being triggered by the stimulus. To guarantee the comparability of the results of the skin conductance and the auto-reports of the perceived pleasantness, we ran supplementary analyses. An analysis including all participants with mean substitutions for missing data gave a similar result *t*_(15)_ = 2.42, *p* = 0.028. Moreover, if we removed the three missing participants from the analysis of the auto reports of pleasantness, the main results did not change *t*_(12)_ = 2.75, *p* = 0.017.

^2^The inspiration before and after for the CS+ was not tested, because sniffing can vary throughout time for several factors (e.g., habituation, see Wirth et al., 1998; or familiarity, see Delplanque et al., 2008). The differential measure (CS+; CS+ similar) is the most appropriate and precise one to test our hypothesis, because it is based on a subtraction that is free of biases related to the time course.
